# Supplementary material for: Geographical distribution of scrub typhus and risk of Orientia tsutsugamushi infection in Indonesia: Evidence mapping
Source: PLoS Negl Trop Dis. 2023 Sep 25;17(9):e0011412. doi: 10.1371/journal.pntd.0011412 (PMC10553813; doi:10.1371/journal.pntd.0011412)
Supplement: S1 Appendix — The full search strategy including search terms and databases searched. (DOCX) [file pntd.0011412.s001.docx]

### Search strategy

#### Databases

1. International databases:
2. Books@Ovid <October 22, 2018>
3. Journals@Ovid Full Text <October 25, 2018>
4. CAB Abstracts <1990 to 2018 Week 42>
5. CAB Abstracts <1910 to 1989>
6. Embase 1974 to present
7. Forest Science <1939 to 2018 Week 42>
8. Global Health <1973 to 2018 Week 42>
9. Medline (Ovid MEDLINE® Epub Ahead of Print, In-Process & Other Non-Indexed Citations, Ovid MEDLINE® Daily and Ovid MEDLINE®) 1946 to present
10. Zoological Record <1978 to 2008>
11. Zoological Record Archive <1864 to 1977>
12. Zoological Record <2009>
13. Scopus
14. IMSEAR database
15. Indonesian databases:
16. <https://garuda.ristekbrin.go.id/>
17. <https://www.neliti.com/id/>
18. ejournal.litbang.depkes.go.id
19. Dutch journals:
20. <https://www.ntvg.nl/>
21. <http://www.ariez.nl/artikelen.1206.lynkx?tijdschrift=tvi&event=showform>
22. <https://dare.uva.nl/>
23. Dutch museums:
24. Natuurhistorisch Museum Rotterdam ([hetnatuurhistorisch.nl/](https://www.hetnatuurhistorisch.nl/))
25. Naturalis Biodiversity Center ([bioportal.naturalis.nl](http://bioportal.naturalis.nl))
26. Libraries:
    1. Eijkman Institute library
27. Local universities and research centres:
    1. Institute of Tropical Disease, Universitas Airlangga
    2. Pusat Kedokteran Tropis (Center of Tropical Medicine), Universitas Gajah Mada
    3. Center for Research and Integrated Development of Tropical Health and Infectious Diseases (CRID-TROPHID), Universitas Indonesia
    4. Others based on the Ministry of Research, Technology, and Higher Education (Kemenristekdikti) list (http://www.dikti.go.id/perguruan-tinggi/)
28. Ministry of Health networks:
    1. Perpustakaan Departemen Kesehatan (Central Health Library)
    2. Perpustakaan Sekretariat Badan Litbangkes (Indonesia NIHRD Secretariat Library)
    3. Litbangkes cabang (Indonesia NIHRD branch):
       1. Balai Besar Litbang Vektor dan Reservoir Penyakit (B2P2VRP) Salatiga
       2. Balai Litbang Pemberantasan Penyakit Bersumber Binatang (P2B2) Donggala
       3. Balai Litbang P2B2 Tanahbumbu
       4. Balai Litbang P2B2 Banjarnegara
       5. Loka Litbang P2B2 Baturaja
       6. Loka Litbang P2B2 Ciamis
       7. Loka Litbang P2B2 Waikabubak

For searches in libraries, local universities, and Ministry of Health networks, search in their websites first (if any). If there is no website, find a contact person.

#### Search language

1. English
2. Dutch: For pre-independence (1945) articles

Search in Indonesian was not done since pilot search has shown that there were many duplicates surfaced as results. All of the relevant articles in Indonesian found during piloting always use/mention “scrub typhus” in the text.

#### Search and cataloguing protocol

1. Login to databases
2. Type the search terms in the search box (refer to specific databases below)
3. Export the search results to EndNote
4. Remove duplicates
5. Screen the title and abstract
6. Make groups in the EndNote library:

- ‘Relevant’: for papers fitting the eligibility criteria
- ‘Useful’: for papers not fitting the eligibility criteria, but providing useful information (e.g. review)
- ‘Excluded’: for papers completely irrelevant

1. Group into: ‘relevant’, ‘useful’, ‘excluded’
2. Retrieve the full text through EndNote
3. Manually search if not found
4. Request inaccessible full text through Bodleian Library, Oxford
5. Review full text
6. Catalogue the relevant papers in EndNote

#### Search protocol Ovid search

1. Select ‘Advanced Search’
2. Run the search in the following databases:
3. Books@Ovid <October 22, 2018>
4. Journals@Ovid Full Text <October 25, 2018>
5. CAB Abstracts <1990 to 2018 Week 42>
6. CAB Abstracts <1910 to 1989>
7. Embase 1974 to present
8. Forest Science <1939 to 2018 Week 42>
9. Global Health <1973 to 2018 Week 42>
10. Medline (Ovid MEDLINE® Epub Ahead of Print, In-Process & Other Non-Indexed Citations, Ovid MEDLINE® Daily and Ovid MEDLINE®) 1946 to present
11. Zoological Record <1978 to 2008>
12. Zoological Record Archive <1864 to 1977>
13. Zoological Record <2009>
14. Search strategy:

1 scrub typhus/ (7543)

2 Orientia tsutsugamushi/ (6243)

3 "scrub typhus".ti,ab. (6948)

4 tsutsugamushi.ti,ab. (6292)

5 scrubtyphus.mp. [mp=tx, bt, ti, ab, ct, ot, hw, id, cc, tn, dm, mf, dv, kw, fx, dq, nm, kf, px, rx, ui, sy] (35)

6 "mite typhus".mp. [mp=tx, bt, ti, ab, ct, ot, hw, id, cc, tn, dm, mf, dv, kw, fx, dq, nm, kf, px, rx, ui, sy] (50)

7 "japanese river fever".mp. [mp=tx, bt, ti, ab, ct, ot, hw, id, cc, tn, dm, mf, dv, kw, fx, dq, nm, kf, px, rx, ui, sy] (111)

8 "kedani fever".mp. [mp=tx, bt, ti, ab, ct, ot, hw, id, cc, tn, dm, mf, dv, kw, fx, dq, nm, kf, px, rx, ui, sy] (9)

9 "chigger borne rickettsia".mp. [mp=tx, bt, ti, ab, ct, ot, hw, id, cc, tn, dm, mf, dv, kw, fx, dq, nm, kf, px, rx, ui, sy] (0)

10 "chigger borne rickettsiosis".mp. [mp=tx, bt, ti, ab, ct, ot, hw, id, cc, tn, dm, mf, dv, kw, fx, dq, nm, kf, px, rx, ui, sy] (30)

11 "chigger borne typhus".mp. [mp=tx, bt, ti, ab, ct, ot, hw, id, cc, tn, dm, mf, dv, kw, fx, dq, nm, kf, px, rx, ui, sy] (4)

12 "mite fever".mp. [mp=tx, bt, ti, ab, ct, sh, ot, hw, id, cc, tn, dm, mf, dv, kw, fx, dq, nm, kf, px, rx, an, ui, ds, on, sy] (51)

13 "mite borne rickettsia".mp. [mp=tx, bt, ti, ab, ct, ot, hw, id, cc, tn, dm, mf, dv, kw, fx, dq, nm, kf, px, rx, ui, sy] (8)

14 "mite borne rickettsiosis".mp. [mp=tx, bt, ti, ab, ct, ot, hw, id, cc, tn, dm, mf, dv, kw, fx, dq, nm, kf, px, rx, ui, sy] (27)

15 "mite borne typhus".mp. [mp=tx, bt, ti, ab, ct, ot, hw, id, cc, tn, dm, mf, dv, kw, fx, dq, nm, kf, px, rx, ui, sy] (96)

16 1 or 2 or 3 or 4 or 5 or 6 or 7 or 8 or 9 or 10 or 11 or 12 or 13 or 14 or 15 (10747)

17 trombiculid*.mp. [mp=tx, bt, ti, ab, ct, ot, hw, id, cc, tn, dm, mf, dv, kw, fx, dq, nm, kf, px, rx, ui, sy] (7230)

18 leptotrombidium.mp. [mp=tx, bt, ti, ab, ct, ot, hw, id, cc, tn, dm, mf, dv, kw, fx, dq, nm, kf, px, rx, ui, sy] (2624)

19 chigger.mp. [mp=tx, bt, ti, ab, ct, ot, hw, id, cc, tn, dm, mf, dv, kw, fx, dq, nm, kf, px, rx, ui, sy] (2745)

20 17 or 18 or 19 (8780)

21 indonesia.mp. [mp=tx, bt, ti, ab, ct, ot, hw, id, cc, tn, dm, mf, dv, kw, fx, dq, nm, kf, px, rx, ui, sy] (128821)

22 "Netherlands East Indies".mp. [mp=tx, bt, ti, ab, ct, ot, hw, id, cc, tn, dm, mf, dv, kw, fx, dq, nm, kf, px, rx, ui, sy] (298)

23 "Dutch East Indies".mp. [mp=tx, bt, ti, ab, ct, ot, hw, id, cc, tn, dm, mf, dv, kw, fx, dq, nm, kf, px, rx, ui, sy] (1449)

24 "Malay archipelago".mp. [mp=tx, bt, ti, ab, ct, ot, hw, id, cc, tn, dm, mf, dv, kw, fx, dq, nm, kf, px, rx, ui, sy] (1409)

25 "Dutch New Guinea".mp. [mp=tx, bt, ti, ab, ct, ot, hw, id, cc, tn, dm, mf, dv, kw, fx, dq, nm, kf, px, rx, ui, sy] (401)

26 "Netherlands New Guinea".mp. [mp=tx, bt, ti, ab, ct, ot, hw, id, cc, tn, dm, mf, dv, kw, fx, dq, nm, kf, px, rx, ui, sy] (361)

27 21 or 22 or 23 or 24 or 25 or 26 (131037)

28 16 or 20 (17229)

29 27 and 28 (330)

1. Export to EndNote

#### Search protocol for Global Index Medicus

1. Search strategy:

tw:((("scrub typhus" OR tsutsugamushi OR scrubtyphus OR "mite typhus" OR "japanese river fever" OR "kedani fever" OR "chigger borne rickettsia" OR "chigger borne rickettsiosis" OR "chigger borne typhus" OR "mite fever" OR "mite borne rickettsia" OR "mite borne rickettsiosis" OR "mite borne typhus") OR (trombiculid* OR leptotrombidium OR chigger)) AND (indonesia OR "Netherlands East Indies" OR "Dutch East Indies" OR "Malay archipelago" OR "Dutch New Guinea" OR "Netherlands New Guinea")) AND (instance:"ghl") AND ( db:("IMSEAR"))

1. Select ‘IMSEAR (SEARO)’ under the ‘Sources’ drop down list

#### Search protocol for Scopus

- 1. Search strategy:

( ( ( TITLE-ABS-KEY ( "scrub typhus" ) OR TITLE-ABS-KEY ( tsutsugamushi ) OR TITLE-ABS-KEY ( scrubtyphus ) OR TITLE-ABS-KEY ( "mite typhus" ) OR TITLE-ABS-KEY ( "japanese river fever" ) OR TITLE-ABS-KEY ( "kedani fever" ) OR TITLE-ABS-KEY ( "chigger borne rickettsia" ) OR TITLE-ABS-KEY ( "chigger borne rickettsiosis" ) OR TITLE-ABS-KEY ( "chigger borne typhus" ) OR TITLE-ABS-KEY ( "mite fever" ) OR TITLE-ABS-KEY ( "mite borne rickettsia" ) OR TITLE-ABS-KEY ( "mite borne rickettsiosis" ) OR TITLE-ABS-KEY ( "mite borne typhus" ) ) ) OR ( ( TITLE-ABS-KEY ( trombiculid* ) OR TITLE-ABS-KEY ( leptotrombidium ) OR TITLE-ABS-KEY ( chigger ) ) ) ) AND ( ( TITLE-ABS-KEY ( indonesia ) OR TITLE-ABS-KEY ( "Netherlands East Indies" ) OR TITLE-ABS-KEY ( "Dutch East Indies" ) OR TITLE-ABS-KEY ( "Malay archipelago" ) OR TITLE-ABS-KEY ( "Dutch New Guinea" ) OR TITLE-ABS-KEY ( "Netherlands New Guinea" ) ) )

#### Search protocol for Indonesian websites

1. Login if search information can be saved
2. Type the search terms in the search box:

- “scrub typhus”
- tsutsugamushi
- leptotrombidium

1. If possible, export the search results (citation and abstract) to EndNote. If not, download the pdf and enter manually into EndNote.

#### Search protocol for Dutch websites

- 1. The listed Dutch websites does not seem to return relevant search results with long search string.
  2. Type the following search terms:
- “scrub typhus”
- scrubtyfus
- “scrub tyfus”
- mijtekoorts
- tsutsugamushi
- trombiculid
- leptotrombidium
- chigger
  1. Screen for relevant articles
