# Supplementary material for: Geographical distribution of scrub typhus and risk of Orientia tsutsugamushi infection in Indonesia: Evidence mapping
Source: PLoS Negl Trop Dis. 2023 Sep 25;17(9):e0011412. doi: 10.1371/journal.pntd.0011412 (PMC10553813; doi:10.1371/journal.pntd.0011412)
Supplement: S3 Appendix — A Word document with additional information on methods and results: (1) Section A. Ranking and scores of evidence based on the likelihood of sustaining infectious vectors and recency; (2) Section B. Criteria of vectorship; (3) Fig A. The scoring algorithm applied to score each subnational unit; (4) Fig B. The number of data points collection ending in each period; (5) Fig C. The distribution of data points across the subnational units in Indonesia; (6) Table A. The distribution of data points with seropositive participants across subnational units; (7) Table B. The number of data points for each non-human host species; (8) Table C. The distribution of data points on vector presence across subnational units; (9) Table D. Scores and number of data points in each subnational unit. (DOCX) [file pntd.0011412.s003.docx]

1. **Ranking and scores of evidence based on the likelihood of sustaining infectious vectors and recency**

The ranking of evidence and scores in detail are below (Figure A):

1. Confirmed case of human scrub typhus (score +24)
2. Probable case of human scrub typhus (score +22)
3. Possible case of human scrub typhus, diagnosed by Weil-Felix test (score +19)
4. Possible case of human scrub typhus, diagnosed clinically (score +18)
5. Seroprevalence of >20% in community (score +16)
6. Seroprevalence of >10% and ≤20% in the community (score +15)
7. Seroprevalence of >5% and ≤10% in the community (score +14)
8. Seroprevalence of 5% or less (but >0) in the community (score +13)
9. Infection confirmed in vector (score +10)
10. Infection confirmed in other hosts and presence of vector (score +8)
11. Infection confirmed in other hosts but presence of vector is unknown (score +6)
12. Presence of vector but presence of *O. tsutsugamushi* is unknown (score +3)

In addition, all evidence of presence gathered were given a score based on the year when sample collection or observation ended i.e. study end year:

1. 1945 or older: 0
2. 1946 to 1965: score +1
3. 1966 to 1985: score +2
4. 1986 to 2005: score +3
5. 2005 to present: score+4

We used the study end year because it was more representative than the publication date.

1. **Criteria of vectorship**

‘Criteria of vectorship’ [1]: (1) the candidate vector needs to be proven naturally infected by *O. tsutsugamushi*; (2) there must be evidence that the mite species can transmit the pathogen to a host; (3) the species need to be present in the areas when scrub typhus is prevalent; (4) naturally infected human hosts should also be present where scrub typhus is prevalent; (5) the candidate mites also feed on humans; (6) the species should be able to acquire pathogen from one host and transmit it to another.


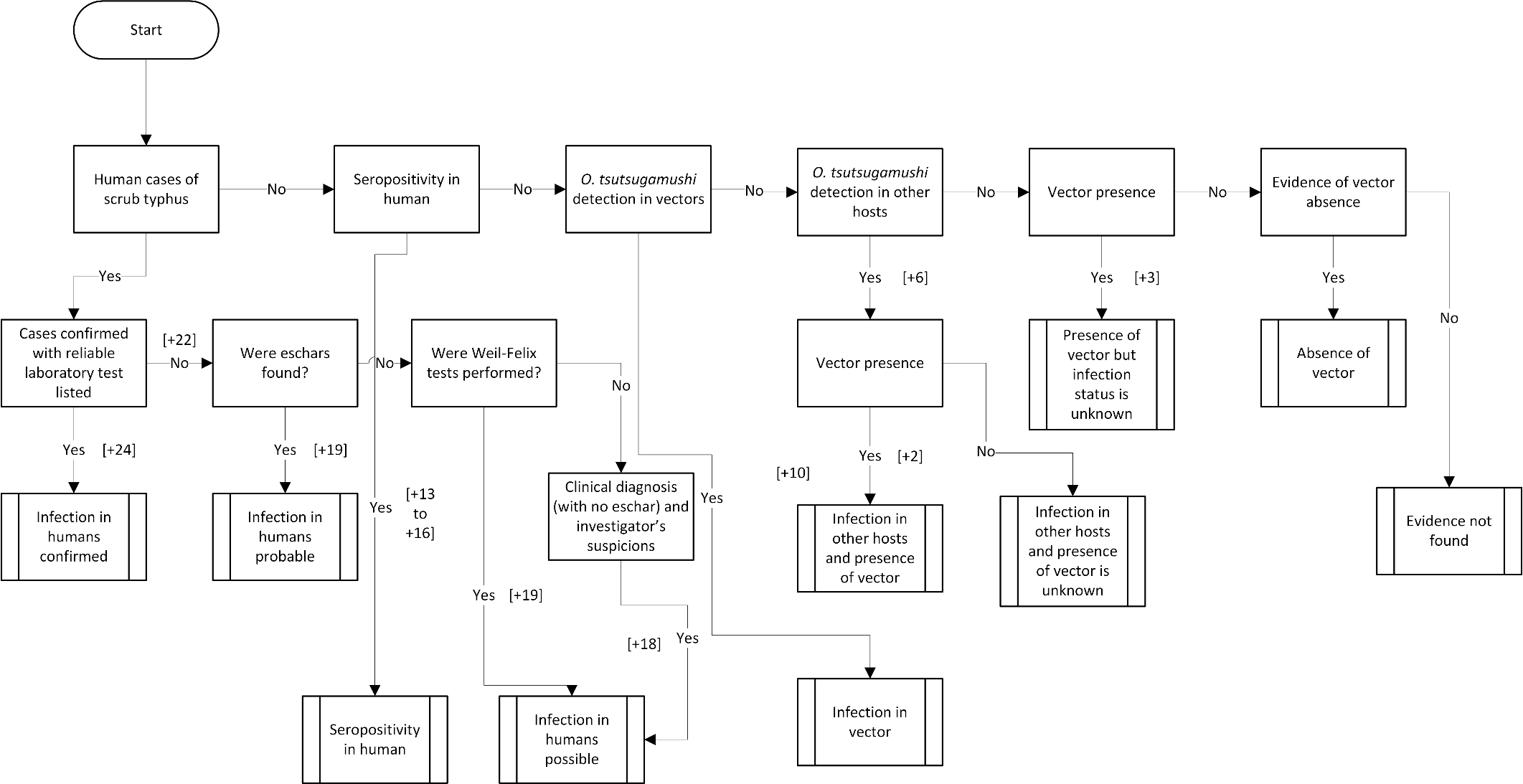


Fig A. The scoring algorithm applied to score each subnational unit


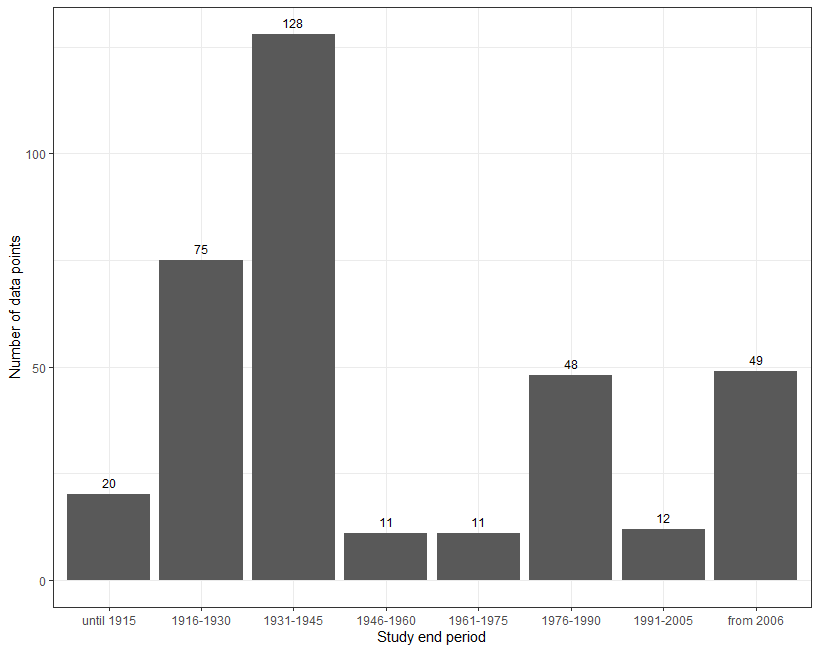


Fig B. The number of data points collection ending in each period


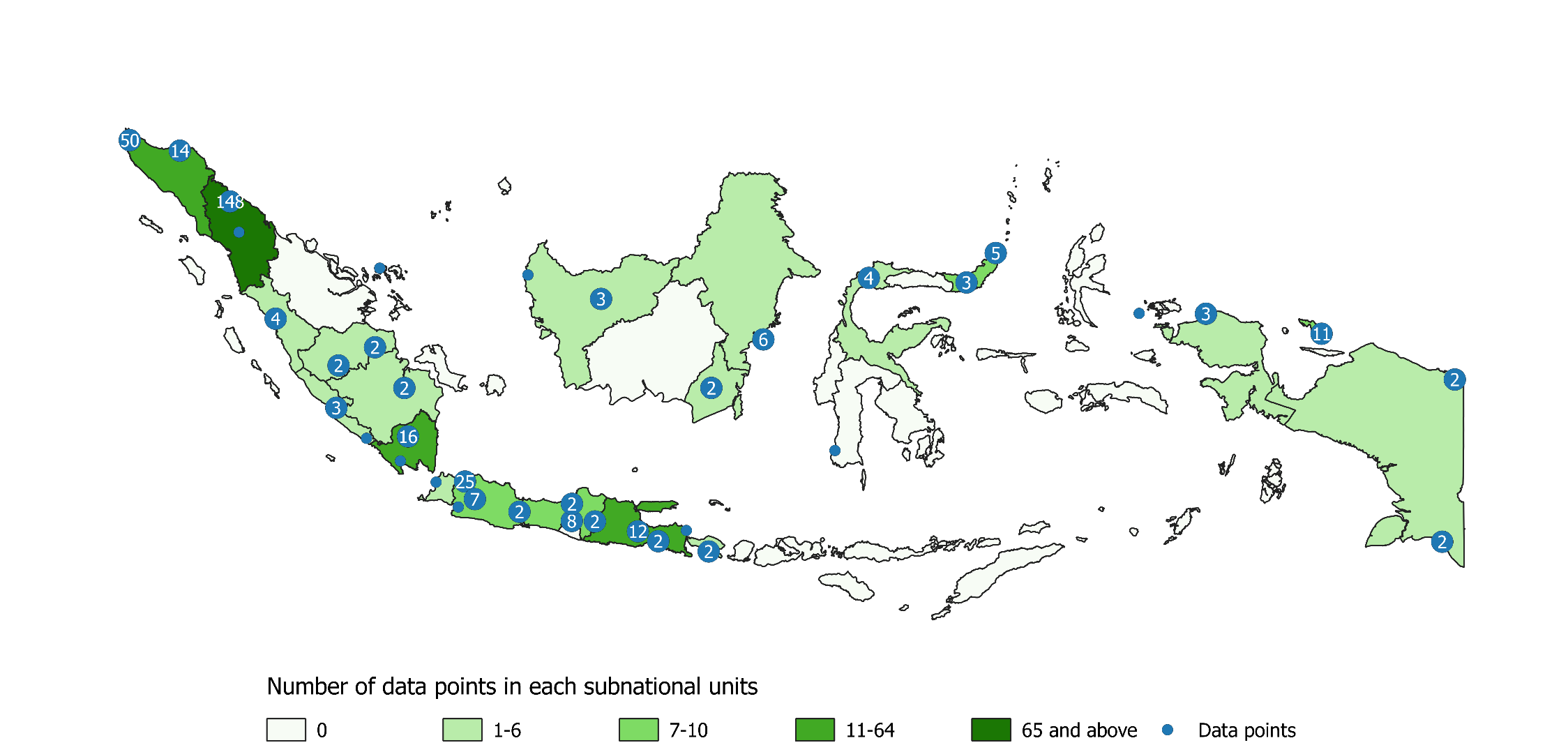


Fig C. The distribution of data points across the subnational units in Indonesia

The colour of the subnational units represented the total number of data points within the boundaries of each subnational unit. The blue dots represented each data point, and if there are any overlapping data points, these were displayed as a cluster (a blue dot with the number of overlapping data points). The number of data points in each subnational units were classified using natural breaks (Jenks). Natural breaks minimise variance within classes by grouping similar values, while emphasising the differences between classes [2, 3]. The range of the number of data points from each subnational unit was 1 – 169 and the median was 4, which could be considered as a wide range with most of the subnational units having way less than 169 data points (22 out of 25 (88.0%) of the subnational units had less than 20 data points). Natural breaks can present the wide range clearly while still providing contrasts between the classes of subnational units with smaller number of data points. The boundaries shown in this map were obtained from the UN Food and Agriculture Organisation’s Global Administrative Unit Layers [4] as modified by Moyes et al [5].

Table A. The distribution of data points with seropositive participants across subnational units

| **Subnational unit** | **Number of data point(s)** | **Percentage** | **Seroprevalence (%)** |
| --- | --- | --- | --- |
| East Java | 4 | 21.1 | 0.63, 1.3, 3.65, 7.88 |
| Lampung | 4 | 21.1 | 5.7, 6.6, 11.9, 20 |
| Bali | 2 | 10.5 | 7.95, 8.96 |
| Jakarta | 2 | 10.5 | 0.36, 3.2 |
| Waigeo+ | 1 | 5.3 | 9.4 |
| West Java | 1 | 5.3 | 9.3 |
| Jambi | 1 | 5.3 | 9.71 |
| Yogyakarta | 1 | 5.3 | 6 |
| Papua | 1 | 5.3 | 4.42 |
| South Sulawesi | 1 | 5.3 | 2.9 |
| Central Java | 1 | 5.3 | 2 |

Table B. The number of data points for each non-human host species

| **Host** | **Number of data point(s)** | **Percentage** |
| --- | --- | --- |
| *Rattus tanezumi* | 7 | 21.2 |
| *Rattus tiomanicus* | 4 | 12.1 |
| *Rattus rattus* | 4 | 12.1 |
| *Rattus exulans* | 3 | 9.1 |
| *Rattus argentiventer* | 3 | 9.1 |
| *Rattus norvegicus* | 2 | 6.1 |
| Rodents | 2 | 6.1 |
| *Bandicota indica* | 1 | 3.0 |
| *Chiropodomys gloroides* | 1 | 3.0 |
| *Maxomys sp* | 1 | 3.0 |
| *Maxomys surifer* | 1 | 3.0 |
| *Niviventer fulvescens* | 1 | 3.0 |
| *Leopoldamys sabanus* | 1 | 3.0 |
| *Maxomys whiteheadi* | 1 | 3.0 |
| *Rattus sp* | 1 | 3.0 |
| Total | 33 | 100.0* |

*Three data points used more than one diagnostic method (isolation and IFA), therefore, the sum of all the previously mentioned percentage is more than 100%.

Table C. The distribution of data points on vector presence across subnational units

| **Subnational unit** | **Number of data point (s)** | **Percentage** |
| --- | --- | --- |
| Jakarta | 10 | 13.5 |
| West Java | 9 | 12.2 |
| Central Java | 9 | 12.2 |
| East Java | 9 | 12.2 |
| Lampung | 6 | 8.1 |
| Biak | 5 | 6.8 |
| North Sumatra | 4 | 5.4 |
| Bengkulu | 3 | 4.1 |
| Jambi | 3 | 4.1 |
| Papua | 3 | 4.1 |
| North Sulawesi | 3 | 4.1 |
| South Borneo | 2 | 2.7 |
| Central Sulawesi | 2 | 2.7 |
| Banten | 1 | 1.4 |
| Bintan+ | 1 | 1.4 |
| East Borneo | 1 | 1.4 |
| Aceh | 1 | 1.4 |
| West Papua | 1 | 1.4 |
| South Sumatra | 1 | 1.4 |
| Total | 74 | 100.0 |

Table D. Scores and number of data points in each subnational unit

| **Subnational unit** | **Score** | **Number of data points** |
| --- | --- | --- |
| Biak | 23 | 10 |
| South Sumatra | 23 | 3 |
| North Sumatra | 22 | 149 |
| Aceh | 22 | 64 |
| Jakarta | 22 | 23 |
| West Borneo | 22 | 4 |
| Bengkulu | 22 | 4 |
| West Papua | 22 | 3 |
| Banten | 22 | 2 |
| West Java | 18 | 10 |
| Jambi | 18 | 4 |
| Bali | 18 | 2 |
| Yogyakarta | 18 | 1 |
| East Java | 17 | 17 |
| Lampung | 17 | 16 |
| South Sulawesi | 17 | 1 |
| Waigeo+ | 17 | 1 |
| Papua | 16 | 5 |
| North Sulawesi | 14 | 8 |
| East Borneo | 14 | 6 |
| West Sumatra | 14 | 4 |
| Central Sulawesi | 12 | 4 |
| Central Java | 7 | 10 |
| South Borneo | 5 | 2 |
| Bintan+ | 5 | 1 |

**References**

1. Traub R, Wisseman CL, Jr. The ecology of chigger-borne rickettsiosis (scrub typhus). J Med Entomol. 1974;11(3):237-303. doi: 10.1093/jmedent/11.3.237.

2. de Smith M, Goodchild M, Longley P. Classification and clustering. 2020. In: Geospatial Analysis [Internet]. 6^th^ edition. Available from: https://www.spatialanalysisonline.com/HTML/index.html.

3. ESRI. Data classification methods. 2022 [Cited 20 January 2022] Available from: https://pro.arcgis.com/en/pro-app/2.7/help/mapping/layer-properties/data-classification-methods.htm.

4. Food Agriculture Organization of the United Nations. The Global Administrative Unit Layers (GAUL): Technical aspects. Food and Agriculture Organization of the United Nations, EC-FAO Food Security Program; 2008.

5. Moyes CL, Henry AJ, Golding N, Huang Z, Singh B, Baird JK, et al. Defining the geographical range of the *Plasmodium knowlesi* reservoir. PLoS Negl Trop Dis. 2014;8(3):e2780. doi: 10.1371/journal.pntd.0002780.
